# Implementation of human genetic counseling in the MZEB (Medical Center for Adults with disabilities) at the psychiatric LVR Clinic Bedburg-Hau

**DOI:** 10.1515/medgen-2024-2026

**Published:** 2024-06-06

**Authors:** Dorothee Maliszewski-Makowka, Dagmar Wieczorek

**Affiliations:** LVR Clinic Bedburg-Hau Bahnstr.6 47551 Bedburg-Hau Germany; Institute of Human Genetics Medical Faculty and University, Hospital Dusseldorf Moorenstr. 5 40225 Dusseldorf Germany

**Keywords:** MZEB, genetic counselling, psychiatric illnesses, intellectual disability, adults

## Abstract

Outpatient diagnostics for adult patients with intellectual disabilities and developmental disorders were significantly improved when ‘Medical Centers for Adults with Disabilities’ (MZEB) were established in 2015 in accordance with a new law (§ 119c SGB V). Due to the multi-professional nature of these MZEBs, cooperation with various specialized centers can be initiated. Accordingly, in 2023, a cooperation between the MZEB in the LVR-Clinic Bedburg-Hau and the Institute of Human Genetics, Heinrich-Heine University (HHU) Düsseldorf was initiated. Interdisciplinary consultation hours for adult patients have been established in Bedburg-Hau offering genetic counselling and testing to identify the underlying genetic entity. We will introduce this new structure and report preliminary results.

## Prior to our cooperation, genetic testing for adults with mental disabilities and psychiatric illnesses was rarely carried out, not being part of standard care

At the treatment center for inclusive medicine at the LVR-Clinic Bedburg-Hau, which until 2017 consisted of only a psychiatric inpatient ward and a psychiatric outpatient clinic, the importance of incorporating more accessible genetic counselling and testing into the patient care paradigm has long been acknowledged. Despite the potential benefit these examinations provide for the patient, their realization faced various difficulties.

In our psychiatric inpatient wards, an indication for genetic testing was discussed with the patients, but frequently it was not possible to arrange this. One of the reasons was the expensiveness of these examinations, which are not financed by the German health insurance system as part of inpatient treatment. Additionally, genetic testing entails significant organizational and time expenditure, requiring a consultation, an appointment, and appropriate motivation from those affected. As such, preparation and implementation of the examinations went beyond the scope of inpatient treatment.

Moreover, especially in acute treatment, those affected could only rarely be motivated to pursue genetic examinations related to their current ailment. The results of the genetic examination would only be available at a much later point in time, long after the patient had been discharged from the inpatient setting, discouraging the patients and making it difficult to organize a subsequent interdisciplinary discussion as well as explanation of the respective findings.

While genetic examinations are financed in ambulatory care by the German health insurance system, the resources of the providers in our psychiatric outpatient clinic were typically even more insufficient to conduct genetic counselling and associated motivational conversations.

Another problem is the patients rarely being accompanied by their legal guardian in person during their visits in the ambulatory clinic. Similarly, communication during residential home visits mostly takes place with the patient and nursing staff, and not directly with the legal guardian. In both scenarios legal guardians are briefed on test outcomes and suggested treatment plans in separate follow-up discussions, which, considering the necessity of usually several motivational interviews with both the patient and their legal guardian, significantly extends the available time and resources.

This made genetic counselling difficult to establish in our standard outpatient care, despite the comparably high interest among the ambulatory patients with their frequently more chronic conditions.

Now, however, the introduction of the MZEBs removes many obstacles for implementation of interdisciplinary measures, allowing us to include human genetic counseling and testing more regularly into our patient care. The first preliminary results of this new structure are presented here.

## The mission of the MZEBs

The ‘Medical Centers for Adults with Disabilities’ (MZEBs) aim to provide needs-based medical care in the form of an interdisciplinary and multi-professional service if and for as long as the severity or complexity of the disability or disability-related health problems exceed the possibilities of the standard medical care system for adults with multiple disabilities.

The MZEBs are essential if the upstream levels of care, such as the general practitioner or specialist care, cannot meet the professional requirements. A person with a disability can make use of a MZEB on a temporary or long-term basis, as well as simultaneously or subsequently with mainstream care, provided this is appropriate and meets their needs.

The multi-professional team and equipment of the MZEBs makes it possible to create individual settings for diagnostics and therapy for patients with multiple disabilities, while taking into account the special needs for detailed and disability-friendly communication about the condition on all levels with the patients, legal guardians, and aides involved.

To ensure the effective diagnosis and treatment of this demographic, it is essential that they receive care from physicians and therapists with expertise tailored to their unique needs, encompassing appropriate knowledge, communication, and action. For quality assurance purposes, staff at the MZEB are obliged to undergo consistent training in this domain and provide additional education for involved aides.

The legal basis for the MZEBs is Section 119c SGB V (Social Security Code), which regulates the approval of an MZEB to participate in SHI-accredited medical care (“entitlement of persons with statutory health insurance to medical, dental and psychotherapeutic treatment”). Section 43b SGB V is supplemented as follows: “Insured adults with mental disabilities or severe multiple disabilities are entitled to non-medical services, in particular psychological, therapeutic and psychosocial services, if they are provided under medical responsibility by a medical care center in accordance with Section 119c and are necessary to identify an illness as early as possible and to draw up a treatment plan”.

The respective MZEBs have specialized in different areas. For example, there are MZEBs with an orthopedic, neurological, or internal medicine focus. Treatment at the MZEB should not be permanent. As a rule, it involves extensive diagnostics that cannot be guaranteed to the required extent by the structures of standard care. Once the detailed and complex diagnostics have been completed, a treatment plan or treatment recommendation is drawn up. This is communicated in written and verbal form with all parties involved (patients, legal representatives, physicians providing further treatment, caregivers in the outpatient sector and residential groups) and evaluated at regular intervals. Once this diagnostic phase has been completed and the proposed treatment has been initiated and sufficiently tested for effectiveness, the patients are discharged from treatment in the MZEB to regular treatment.

Each MZEB concludes an individual agreement with the health insurance associations on the provision, remuneration and invoicing of services in accordance with § 119c and § 120 SGB V, removing potential financial issues.

The access criteria and the tasks of the respective MZEB in Germany are defined individually based on the framework concept of the MZEB of the professional associations for people with disabilities on October 12, 2015.

## Organization of the MZEB at the LVR-Clinic Bedburg-Hau

Since July 1, 2017, the treatment center for inclusive medicine at the LVR Clinic Bedburg-Hau has been expanded to include the MZEB and currently consists of a protected inpatient psychiatric ward, the MZEB, and the psychiatric outpatient clinic.

At the LVR-Clinic Bedburg-Hau, a large psychiatric facility, the focus of this MZEB is on diagnosing and drawing up treatment plans for individuals with mental impairments and psychiatric illnesses. The task of our MZEB is to provide comprehensive diagnostics for adults with mental impairments and psychiatric illnesses, which cannot be provided to the required extent under the conditions of standard care. Treatment usually lasts three to four quarters.

Treatment at the MZEB in Bedburg Hau, as an MZEB with a psychiatric focus, is only possible under certain criteria (see Table 1).

**Tab. 1:** 

The admission criteria for the MZEB at the LVR Clinic Bedburg-Hau are as follows:
– Completed 18th year of age
– Severely disabled person’s pass with a degree of disability (GdB) of at least 70
– Requirement of target group-specific diagnostics and therapy, in particular specialized communication through suitable communication strategies (simple images, communication aids, assistance)
– Suspected or already diagnosed congenital or acquired degenerative, inflammatory or vascular disease of the nervous or neuromuscular system corresponding to one of the following eligible diagnoses:
– F06 (other mental disorder due to brain damage or dysfunction or physical illness); F07 (personality and behavioral disorder due to brain disease, damage or dysfunction);
– F70–79 (intelligence disorders);
– F80–F89 (developmental disorders);
– G10–G14 (system atrophies that predominantly affect the central nervous system);
– G71 (primary myopathies);
– G80–83 (cerebral palsy and other paralysis syndromes); Q00-Q99 (congenital malformations, deformities and chromosomal abnormalities);
– and additionally R13 (Dysphagia) and R47 (Speech and language disorders not elsewhere classified)

There are access criteria that apply to all MZEBs in Germany; however, individual MZEBs would have to conclude local contracts with the relevant health insurance funds, as they have different specialist focuses.This results from a higher treatment cost compared to regular outpatient treatment. One access criterion for all centers for special education and counseling (MZEBs) is the requirement of having a severe disability rating (GdB) of at least 70.

There must be a need for target group-specific diagnostics and therapy, in particular specialized communication through suitable communication strategies, e. g. simple images, means of communication, assistance.

## Tasks of the MZEB in Bedburg-Hau:

The first step is to summarize the medical findings from the patient’s medical history and take a detailed medical history. This is followed by supplementing the diagnosis with currently or previously missing aspects, by, for example, specialized psychological testing and the collection of assessments, e. g. ICF (International Classification of Functioning, Disability and Health) and SEED (Emotional Development Scale). Based on this detailed examination, a final diagnosis and treatment recommendation is made in accordance with the current S2k guidelines (AWMF Registration number 028-042). The treatment recommendation is communicated with the outpatient physicians responsible for the patient and implemented accordingly following their approval. Afterwards, the patient is discharged from the MZEB.

As mentioned above, in most cases of adults with intellectual development disorders referred to us, the causes of their condition are unknown. It is therefore important to supplement our diagnostics with a human genetic examination to determine or rule out a genetic syndrome.

## Indications for human genetic testing in individuals with intellectual disability

In our view, every patient with an intellectual development disorder, including those with positive family history, consanguinity, associated malformations, facial dimorphisms, should be offered genetic testing in order to accurately diagnose a (non)-syndromic disorder:

There is still a gap between the demands and requirements on one hand side and the reality of the situation on the other side regarding the health care of mentally impaired people with and without psychiatric disorders ([1–8] Fegert et al. 2017, Häßler et al. 2019). Population-based estimates suggest that between 10 and 60 % of all children and adolescents with intellectual disability experience mental disorders, with the higher prevalences in younger children ([9] Munir 2016).

However, identification of causative variants in individuals with intellectual disability and mental impairment is lower than 50 % after short-read exome or genome sequencing ([10] Grozeva et al., 2015, [11] Vissers et al., 2016, [12] Bertoli-Avella, 2021, [13] Mainali et al., 2023)

## Motivation of the patients concerned and their legal representatives with regard to genetic testing

In our experience, patients and their legal representatives often lack awareness regarding the applications of human genetics. Below are the key arguments we present during motivational conversations in our consultations to underscore the potential benefits of genetic testing:

Clarifying the genetic basis of the condition can be an enormous relief for families. In many cases, there are different assumptions about the origin of the condition and associated feelings of guilt on the part of the parents, all of which could be potentially relieved.
Better assessment of the possible course of the disease with a focus on possible somatic risks, enabling targeted preventive measures if possible.
Possibility of participating in appropriate forums and support groups, which can provide valuable support for those affected and their relatives in individual cases.
Prognostic assessment of a hereditary disease.
Finding and prognostic insights concerning treatable conditions.

In conversations about human genetic testing with affected individuals, we have observed various reactions. Frequently, patients and their relatives show initial interest in genetic testing, but many exhibit hesitation when it comes to actually undergoing the test, expressing a desire to take time to consider it further.

After reconsidering, some patients decline, often due to the fact that they may have to travel long distances to the test site and due to the requirement of additional appointments. Occasionally, the caregivers or nursing staff in the residential homes of patients are not prepared to provide organizational support for such an examination, partly because they do not see the need for it, (“Mr/Mrs XY does not wish to have children”). Hence, we have recognized the significant impact that caregivers, relatives, and legal guardians have in guiding the decisions of the individuals under their care or supervision. Therefore, discussions regarding genetic counselling often have to be held on several levels before an examination appointment can finally be arranged.

Ultimately, in most instances, the people concerned are skeptical about the consequences of a human genetic test and expect little subsequent therapeutic intervention.

Organizing a human genetic test close to the patient’s home reduces some of the hurdles and makes it easier for the patient to decide to undergo the test.

## Organization of human genetic counseling and testing as part of the cooperation between the LVR Clinic Bedburg-Hau and the Institute of Human Genetics at Heinrich Heine University in Düsseldorf

The patient’s consultation and examination are initially prepared in cooperation with the Institute of Human Genetics in Düsseldorf and the Center for Inclusive Medicine in Bedburg-Hau. The psychiatric patients with intellectual disabilities visiting the MZEB in Bedburg-Hau are offered genetic counselling and testing. If the patients and their legal guardians agree, they get an appointment for the interdisciplinary consultation hours which take place once a quarter. Some of the individuals prefer to visit the Institute of Human Genetics at the University Hospital Düsseldorf.

The consultation and clinical examination of the patient takes place in presence of their legal guardian or after consent of the legal guardian, the responsible psychiatrist and the human geneticist. The further course of action is determined based on diagnostic tests already performed and initiated after genetic counseling of the patient and the legal guardians. Usually, exome sequencing will be performed after normal karyotyping and normal testing for fragile X syndrome. The results of the examination and the resulting recommendations, including investigations of family members, are communicated to the patients and their legal representatives in the same setting.

## Preliminary results of genetic testing

The human genetic test was proposed to a total of 20 patients. Genetic testing did not take place in 5 patients. One patient discontinued treatment at the MZEB. One patient declined the human genetic examination because his residential group was unable or unwilling to organize the appointments. Three patients refused the examination from the outset without giving reasons or showed no interest in the examination.

To date, 15 patients with ID and various psychiatric diagnoses, e. g. emotionally unstable personality disorder, disorders from the autism spectrum, delusional disorders, visited our interdisciplinary consultation hours and received genetic counseling and subsequent genetic testing (see Table 2). On average three appointments were necessary.

The following are descriptions of three cases.

## Case report 1

Individual 1 is a 29-year-old female patient with intellectual disability, early childhood autism and reduced pain perception. She has two healthy siblings.

The presentation at the MZEB was necessary because the patient had shown a pronounced change in behavior for about a year. At that time, she had been treated as an inpatient for cholecystitis and had not complained of any pain, despite a very advanced inflammation in the abdomen. After this inpatient treatment, the patent had behaved unusually, felt that she was being watched and reacted aggressively.

We diagnosed a delusional disorder, which we successfully treated with a highly potent neuroleptic (Risperidon).

Chromosome analysis revealed a normal female karyotype (46,XX). Single exome analysis identified a heterozygous, pathogenic variant in the X-linked* DDX3X gene* (c.874C>T, p.Arg292*)*.* In this patient, the diagnosis *DDX3X*-associated neurodevelopmental disorder (Snijders-Blok syndrome) could be established. Parental segregation analysis was not performed.

After genetic counseling of the family, the mother decided not to carry out any further human genetic tests. The patient’s biological father is no longer alive. The patient remains under close psychiatric and General Practitioner Treatment care; the focus is on the delusional symptoms and deficits in endurance and affect regulation, which have since remitted under neuroleptic treatment. Due to reduced pain sensation, regular consultations with the General Practitioner Treatment are necessary; in the past, the patient had not noticed for a while that she had acute cholecystitis. The pathogenic variant in *DDX3X* cannot explain the reduced pain sensation; another causative variant could not be identified.

In general, there is no clinical evidence of a genetic pain insensitivity in the patient, as the neurological findings were unremarkable. It is conceivable that the patient was not able to make herself noticed due to the thought disorder and perceptual disorder present in the psychosis, or was unable to communicate her pain clearly.

**Tab. 2:** 

15 patients were consulted in the interdisciplinary consultation – results:
8 patients – no abnormal findings
4 patients – conspicuous findings
2 patients – findings not yet available
1 patient – decided against the examination

The patient’s mother and stepfather were very satisfied with the overall outcome of the consultation because they were keen to exhaust all diagnostic measures to find out the cause of the patient’s reduced sensitivity to pain.

## Case report 2

Patient 2 is a 26-year-old male patient with behavioral abnormalities, intellectual disability, and apraxia and without any facial dysmorphism. According to the parents, the pregnancy was uneventful. Birth was normal at 39th week of gestation. His psychomotor development had been delayed since early childhood. The patient was able to walk without support at the age of 2.5 years. He spoke his first words at the age of 3 years. He can currently form 2-word sentences. He has a moderate intellectual disability. He has focal, atonic and bilateral tonic-clonic seizures since the age of eight. The patient is being treated with frisium, lamotrigine and valproate. Lissencephaly with pachygyria was noted. In addition, there are behavioral abnormalities with impulse control disorders, impulsive outbursts and aggressive behavior towards others. This led to his exclusion from the sheltered workshop. Similar abnormalities can be observed in his younger brother.

Chromosomal and SNP array analysis did not reveal any abnormalities. The phenotype-based single exome analysis identified a hemizygous, pathogenic variant in the *DCX gene:* c.587G>A, p.Arg196His. Pathogenic or probably pathogenic variants in the *DCX* gene are causally related to *DCX-associated* lissencephaly in boys and men with a wide clinical spectrum.

Segregation analysis in the family is still pending, as is the final consultation.

It is very likely that the brother is also affected and the mother is a carrier of the variant, so the diagnosis also has an impact on the counseling of other family members on the mother’s side with regard to the probability of hereditary recurrence following the X-linked inheritance. The patient remains in intensive psychiatric and neurological treatment although no disease-specific therapy is available.

## Case report 3

Individual 3 is a 21-year-old female patient with intellectual disability, facial abnormalities with up slanting palpebral fissures, short philtrum, microretrognathia and a thin upper lip. Her brother is also affected. The patient attended a special school. A reason for the presentation at the MZEB was massive behavioral problems in the sense of frequent and violent states of agitation with destructive rage and verbal assaults. This unpleasant change occurred in connection with the planned change of residential group. The young patient had been living in a residential group for adolescents for several years, where she had felt familiar and self-confident; she wanted to prevent a change in the living environment. After treatment with low-potency neuroleptics and psychological support, the patient was able to make significant progress.

Chromosomal and CNV analyses did not reveal any anomalies. Phenotype-based single exome analysis revealed two heterozygous variants of unknown significance in the *MED13L gene:* c.5737A>T, p.Ser1913Cys and in the *TRIO* gene*:* c.1368G>A, p.Lys456=. To further assess these two variants, we recommended a segregation analysis of the parents and the similarly affected brother. Nothing is known about the potential clinical signs of the parents and the brother. If one of the heterozygous mutations in the patient is causative for both siblings, one has to discuss germinal mosaicism in the parents. This will be discussed with the patient’s legal guardian at the next appointment; however, there is no longer any contact with the parents and with the brother. As the patient may plan children in the future, segregation analyses might be helpful for recurrence risk and possible prenatal testing.

## Outlook

The cooperation between the MZEB at the LVR-Clinic Bedburg-Hau and the Institute of Human Genetics at the University Hospital in Düsseldorf has been successfully established and the genetic consultation service is very well received by our patients. Furthermore, all new patients admitted to the MZEB are informed in detail about the human genetics consultation and examination service during their admission consultation. Throughout the consultation process, the topic is revisited until the final decision regarding human genetic testing has been made by the patient.

In the families concerned, the results of the examination provide greater clarity about the cause of the intellectual disability, aiding in diagnosis, prognosis, and subsequent care. In cases where no abnormalities were found, it brings relief for the siblings and parents of the affected persons. Conversely, in instances of pathological findings, families were grateful for certainty about the diagnosis and further course of action.

We consider human genetic testing in adult patients with intellectual disabilities to be an enormous enrichment of our diagnostics and find it to be an urgently needed measure in line with current medical standards. The establishment of the MZEBs and the cooperation with the Institute of Human Genetics at Heinrich Heine University in Düsseldorf allows comprehensible integration of these measures into the daily lives of our patients. Our plan is to raise awareness among patients with known early childhood developmental disorders about the option of human genetic testing in all settings available to us and to encourage them to seek it.

## References

[1] Neuhäuser G, Steinhausen H-C, Häßler F, Sarimski K (2013). Intellectual disability. Intellectual disability.

[2] Finucane BM, Ledbetter DH, Vorstman JA (2021). Diagnostic genetic testing for neurodevelopmental psychiatric disorders: closing the gap between recommendation and clinical implementation. Curr Opin Genet Dev 68.

[3] Winterholler M (2018). Medical centers for people with disabilities (MZEB) Improvement in the care landscape for people with intellectual and multiple disabilities through § 119 c SGB V?. Medical centers for people with disabilities (MZEB) Improvement in the care landscape for people with intellectual and multiple disabilities through § 119 c SGB V?.

[4] (2015). https://www.diefachverbaende.de/files/stellungnahmen/2015-10-12-Rahmenkonzeption_MZEB_2015.pdf. https://www.diefachverbaende.de/files/stellungnahmen/2015-10-12-Rahmenkonzeption_MZEB_2015.pdf.

[5] Lindquist Sabine, Klotz Ulrike, Bredel-Geissler Anne, Klafke Anja, Timmermann Kristina, Köhler Wolfgang (2023). Medical care for adults with disabilities: high hurdles for the establishment and operation of treatment centers Dtsch Arztebl 2023. 120(49).

[6] Knopp Cordula, Steiner Robin, Lausberg Eva, von Hoegen Caroline, Busse Sabine, Meyer Robert, Eggermann Katja, Schüler Herdit, Begemann Matthias, Eggermann Thomas, Kurth Ingo, Schulz Jörg B., Elbracht Miriam, Maier Andrea (2022). Genetic (re-)evaluation to optimize the care of adults with intellectual disability;Dtsch Arztebl Int 2022; 119. Genetic (re-)evaluation to optimize the care of adults with intellectual disability;Dtsch Arztebl Int 2022; 119.

[7] Fegert JM, Kölch M, Krüger U (2017). Report on the project: Care for sick children and adolescents in Germany – stocktaking and needs analysis. Report on the project: Care for sick children and adolescents in Germany – stocktaking and needs analysis.

[8] Häßler F, Paeckert J, Reis O (2019). Child and adolescent psychiatric care for children and adolescents with intellectual disability and mental disorders in Germany. Healthcare system doi 10.1055/a-0832-2066.

[9] Munir KM (2016). The co-occurrence of mental disorders in children and adolescents with intellectual disability/intellectual developmental disorder. Curr Opin Psychiatry 29.

[10] Grozeva D, Carss K, Spasic-Boskovic O, Tejada MI, Gecz J, Shaw M (2015). Targeted next-generation sequencing analysis of 1,000 individuals with intellectual disability. Hum Mutat. 2015;36.

[11] Vissers LE, Gilissen C, Veltman JA (2016). Genetic studies in intellectual disability and related disorders. Nat Rev Genet. 2016;17.

[12] Bertoli-Avella AM, Kandaswamy KK, Khan S, Ordonez-Herrera N, Tripolszki K, Beetz C, Rocha ME, Urzi A, Hotakainen R, Leubauer A, Al-Ali R, Karageorgou V, Moldovan O, Dias P, Alhashem A, Tabarki B, Albalwi MA, Alswaid AF, Al-Hassnan ZN, Alghamdi MA, Hadipour Z, Hadipour F, Al Hashmi N, Al-Gazali L, Cheema H, Zaki MS, Hüning I, Alfares A, Eyaid W, Al Mutairi F, Alfadhel M, Alkuraya FS, Al-Sannaa NA, AlShamsi AM, Ameziane N, Rolfs A, Bauer P.Genet Med (2021 Aug). Combining exome/genome sequencing with data repository analysis reveals novel gene-disease associations for a wide range of genetic disorders. ;23(8).

[13] Apurba Mainali, Taryn Athey, Shalini Bahl, Hung Clara, Caluseriu Oana, Chan Alicia, Eaton Alison, Ghai Shailly Jain, Kannu Peter, MacPherson Melissa, Niederhoffer Karen Y, Siriwardena Komudi, Mercimek-Andrews Saadet Diagnostic yield of clinical exome sequencing in adulthood in medical genetics clinics. Diagnostic yield of clinical exome sequencing in adulthood in medical genetics clinics.

